# A Report on IAEA/RCA C7-RAS 6/061-004 Training Course in Chiba, Japan in 2014

**Published:** 2015

**Authors:** Shigeru Kosuda, Tsuneo Saga, Diana Paez

**Affiliations:** 1 Department of Radiology, National Defense Medical College; 2 Molecular Imaging Center, National Institute of Radiological Sciences; 3 Nuclear Medicine and Diagnostic Imaging, Division of Human Health, Department of Nuclear Sciences and Applications, IAEA

**Keywords:** Oncology, PET/CT, SPECT/CT, Training Course

## Abstract

The C7-RAS 6/061-004 training course by the International Atomic Energy Agency/Regional Cooperative Agreement (IAEA/RCA) was held in Chiba in 2014. The syllabus, pre- and post-course evaluations, and survey questionnaire results were assembled in this course. The post-course evaluation, including 32 questions similar to the pre-course evaluation, was performed right after the end of the final educational lecture. The mean score showed an improvement, with the score rising from 57.0 points at the beginning to 66.5 points at the end. Among 22 trainees, the greatest score was in a higher range, with an improvement from 82 points at the beginning to 88 points at the end. The grading distribution, with regard to the training course, was as follows: excellent (68.2%), good (31.8%), average (0%), fair (0%), and poor (0%). This report on the training course, held in Chiba in 2014, will contribute to the future global plans of IAEA/RCA. Continuous training courses in member states are required to decrease the present disparities in the knowledge level, instrumentation, and human resources.

## Introduction

Prevalence of non-communicable diseases, particularly cancer, is increasing, worldwide (1). Hybrid nuclear medicine devices, such as positron emission tomography/computed tomography (PET/CT), single-photon emission computerized tomography/CT (SPECT/CT), and PET/magnetic resonance imaging (PET/MRI) scanners, play an important role in the diagnostic work-up of cancer patients (2).

The majority of member states in Regional Cooperative Agreement (RCA) have already installed cyclotrons, hybrid PET/CT scanners, and SPECT/CT scanners and commenced the use of these new modalities. Moreover, some other member states are in the process of introducing hybrid technologies, mainly PET/CT scanners and cyclotrons, or are planning to apply these technologies in near future.

## Course characteristics

This regional training course, called “Improving Cancer Management with Hybrid Nuclear Medicine Imaging” was held from June 30 to July 4 in 2014 at the National Institute of Radiological Sciences (NIRS) in Chiba, Japan. The course was implemented as part of the RAS/6/061project by the International Atomic Energy Agency (IAEA), entitled “Strengthening Clinical Applications of PET/CT, SPECT/CT and PET/MRI in RCA Member States”. The training course was hosted by NIRS and supported by the Japanese Society of Nuclear Medicine.

The overall objective of the training course was to improve professional knowledge, report the skills of nuclear medicine practitioners, and increase the clinical impact of hybrid imaging. In addition, we attempted to provide participants with the most recent updates on the use of PET/CT, SPECT/CT, and PET/MRI in clinical practice and more specifically in cancer management.

## Content of the training course

**Figure 1:**
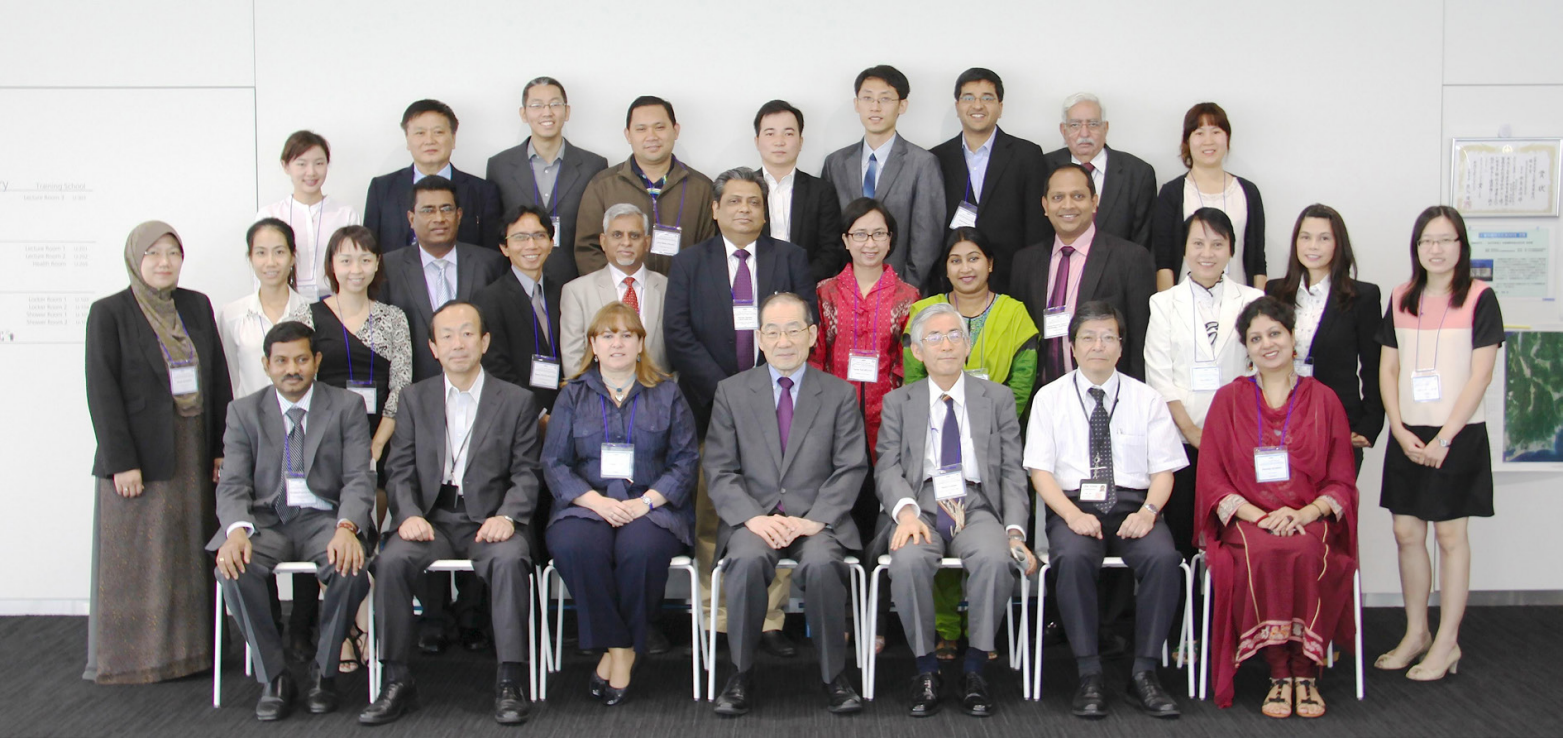
The trainees, lecturers, directors, and NIRS administrators

A total number of 22 trainees from 11 countries and 34 lecturers attended the training course (Figure 1). The number of educational lectures, lunch lectures, and case review sessions was 37, 4, and 2, respectively, with a total of 41 educational seminars during a 5-day training course. Lectures on cross-sectional anatomy were given by diagnostic radiologists. The trainees visited five departments in four institutes for hands-on learning. A syllabus book, including the content of all lectures, was given to each trainee.

To have a quantifiable evaluation parameter for the impact of the training course, pre- and post-evaluations were performed.

The theme of the first session was the fundamentals of hybrid imaging. The titles of lectures included “the quality control of PET/CT and PET quantification”, “fundamentals of PET/CT and SPECT/CT scanners”, “PET radionuclide production by a cyclotron” and “current trends”.

Lectures on the second day were with regard to breast cancer, lymphoma, and gynecological/genitourinary tumors. The lectures on the third day were concerned with lung cancer including scientific excursions. Sessions on the fourth day were on neuroendocrine tumors, head & neck tumors (including theranostics), and peptide receptor radionuclide therapy. The NIRS site tours were held in the afternoon.

The educational course on the final day included the fundamentals and clinical indications of hybrid PET/MRI, sentinel node navigation surgery, recent PET tracers in head and neck cancers, and pitfalls and artifacts in FDG PET/CT.

Many discussions and question and answer sessions were held throughout the training course.

## The post-course evaluation

The post-course evaluation included the same 32 questions as the questions given in the pre-course evaluation right after the end of the final educational lecture. The mean score showed an improvement, with the score rising from 57.0 points at the beginning to 66.5 points at the end. Among 22 trainees, the greatest score was within a higher range at the end of the course, compared to the pre-course evaluation, with an improvement from 82 points at the beginning to 88 points at the end.

The questionnaires, with a total number of 27 questions on the course, were given to 22 trainees. The questionnaire items (five governmental items) and answers were as follows:

1. Was this the only IAEA training course you have attended? Yes: 11, No: 11.
2. Did you receive your travel instructions/air ticket well in advance? Yes: 21, No: 1.
3. Did you have problems or difficulties in applying for or receiving a proper VISA? Yes: 3, No: 18.
4. Did you receive instructions/background documentation for attending the training course well in advance so that you could prepare yourself properly for this course? Yes: 21, No: 1.
5. Were the aims and objectives of the course clearly explained and defined? Yes: 22, No: 0.

**Figure 2:**
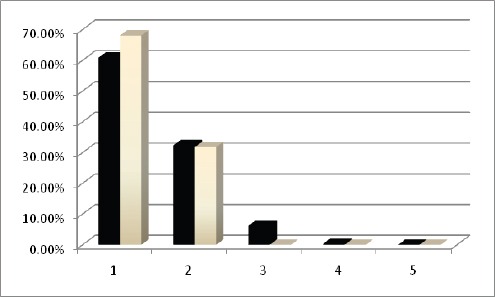
Grading of 21 questions with regard to the training course (black columns) and a general question concerning the training course itself (white columns) is presented as assessed by the trainees. 1=Excellent, 2= Good, 3=Average, 4=Fair, 5=Poor

The answers to 21 questions on the course and one question on the overall rating of the course are shown in Figure 2.

## Discussion

The nuclear medicine section of IAEA has been presenting action plans for approximately 50 years to facilitate and maintain educational training courses. To date, it is estimated that more than 5,000 medical physicians, particularly the younger generation of nuclear medicine specialists, have participated in these regional training courses (3).

In member state countries, disparities have been noted in aspects such as instrumentation, radiopharmaceuticals, and human resources including nuclear medicine technologists, physicists, chemists, pharmacists, and nuclear medicine specialists. Most notably, lack of human resources is a common and significant problem in member state countries. Previous reports have indicated regional heterogeneity in the content, structure, and length of educational training courses on nuclear medicine (3, 4).

All trainees acknowledged that the program was overall well-organized with excellent or proper content (Figure 2). However, some trainees mentioned that the schedule was very tight. Most trainees stated that the course subjects were relevant to their work. Also, as they mentioned, the instructors demonstrated good knowledge, the department staff were cooperative, and the administrators could help all trainees.

As our review indicated, the advantages of this course included well-organized lectures by enthusiastic nuclear medicine experts, brief but informative lectures on cross-sectional anatomy by diagnostic radiologists, hands-on learning in five different facilities, and the syllabus book handed out in the training course. The lectures on cross-sectional anatomy by diagnostic radiologists included all related topics and were accredited by the trainees.

In addition, the number of trainees in diagnostic radiology accounted for one-fourth of all trainees. We think it is important to recommend or invite younger diagnostic radiologists to participate in training courses of nuclear medicine to increase the number of nuclear medicine specialists.

However, the mean score was improved to only 66.5 points at the end. The final score might have improved further if mini-tests were introduced in the interim of the course. Another problem of the training course was lack of budget. Although IAEA supported the training course, the organization had to provide the lecturer fees and travel expenses to domestic lecturers. We express our deepest gratitude to the Japanese Society of Nuclear Medicine and contributing companies for their support of the training course.

Recently, the Asia and Oceania Federation of Nuclear Medicine and Biology (AOFNMB, the president: Henry Hee-Seung Bom) established the Asian Nuclear Medicine Board (ANMB) (5). It is noteworthy that IAEA/RCA training courses and ANMB have aimed at nurturing the younger generation of nuclear medicine specialists. The two educational organizations should communicate and cooperate with each other and compensate for the insufficiencies to achieve greater success in future.

Last but not least, we would like to expect good results and feedbacks brought by applying what the trainees learned during the training course. The organization has to monitor and evaluate the development progress of nuclear medicine in developing countries. It might be necessary to offer some funding, support, and human resources to different countries in order to facilitate the development of nuclear medicine.

We are convinced that the present report on this training course, held in Chiba in 2014, will contribute to the future global planning of IAEA/RCA. Continuous training courses in member state countries are required to decrease the disparities in knowledge, instrumentation, and human resources.
